# SCF^β-TRCP^ targets MTSS1 for ubiquitination-mediated destruction to regulate cancer cell proliferation and migration

**DOI:** 10.18632/oncotarget.1446

**Published:** 2013-11-06

**Authors:** Jiateng Zhong, Shavali Shaik, Lixin Wan, Adriana E. Tron, Zhiwei Wang, Liankun Sun, Hiroyuki Inuzuka, Wenyi Wei

**Affiliations:** ^1^ Department of Pathology, Beth Israel Deaconess Medical Center, Harvard Medical School, Boston, MA; ^2^ Department of Pathophysiology, Norman Bethune College of Medicine, Jilin University, Changchun, P. R. China

**Keywords:** tumor suppressor, MTSS1, ubiquitination, phosphorylation, migration

## Abstract

Metastasis suppressor 1 (MTSS1) is an important tumor suppressor protein, and loss of MTSS1 expression has been observed in several types of human cancers. Importantly, decreased MTSS1 expression is associated with more aggressive forms of breast and prostate cancers, and with poor survival rate. Currently, it remains unclear how MTSS1 is regulated in cancer cells, and whether reduced MTSS1 expression contributes to elevated cancer cell proliferation and migration. Here we report that the SCF^β-TRCP^ regulates MTSS1 protein stability by targeting it for ubiquitination and subsequent destruction via the 26S proteasome. Notably, depletion of either Cullin 1 or β-TRCP1 led to increased levels of MTSS1. We further demonstrated a crucial role for Ser322 in the DSGXXS degron of MTSS1 in governing SCF^β-TRCP^-mediated MTSS1 degradation. Mechanistically, we defined that Casein Kinase Iδ (CKIδ) phosphorylates Ser322 to trigger MTSS1's interaction with β-TRCP for subsequent ubiquitination and degradation. Importantly, introducing wild-type MTSS1 or a non-degradable MTSS1 (S322A) into breast or prostate cancer cells with low MTSS1 expression significantly inhibited cellular proliferation and migration. Moreover, S322A-MTSS1 exhibited stronger effects in inhibiting cell proliferation and migration when compared to ectopic expression of wild-type MTSS1. Therefore, our study provides a novel molecular mechanism for the negative regulation of MTSS1 by β-TRCP in cancer cells. It further suggests that preventing MTSS1 degradation could be a possible novel strategy for clinical treatment of more aggressive breast and prostate cancers.

## INTRODUCTION

Tumor metastasis is a major problem encountered during clinical anti-cancer treatments, which causes higher mortality in cancer patients [1]. Therefore, elucidating the underlying molecular mechanisms that cause tumor growth and metastasis will lead to the development of more effective therapies, in part by eradicating metastatic cancer cells. To this end, it has been established that for many types of human cancers, tumor cells could acquire the capability to metastasize to distant organs that ultimately results in organ failure and death [1, 2]. Although the mechanisms remain largely unknown, overexpression of certain oncoproteins [3] or downregulation of tumor suppressor proteins [4] have been demonstrated to play important roles in the process of tumor growth and metastasis. In this regard, the metastasis suppressor 1 (MTSS1) protein, which is also known as MIM (missing in metastasis) has been recently characterized as a tumor suppressor protein [5]. Notably, MTSS1 is mostly expressed in normal tissues and in some non-metastatic cancer cell lines, however its expression is significantly decreased or mostly absent in many metastatic cancers including metastatic bladder cancer [6], prostate cancer [7], gastric cancer [8] and kidney cancer [9], suggesting that MTSS1 could function as an anti-metastatic protein. Furthermore, an inverse correlation was also observed between MTSS1 expression and poor prognosis in breast cancer [10]. Specifically, findings from large cohort breast cancer clinical samples indicated that decreased MTSS1 expression was positively associated with poorer prognosis, whereas high levels of MTSS1 correlated with an increased overall patient survival [10]. Interestingly, it was reported that all three MTSS1 splice variants were significantly reduced in prostate cancer, whereas overexpression of MTSS1 markedly reduced the proliferation of prostate cancer cells [7]. These findings indicate that MTSS1 might function as a tumor suppressor, and loss of MTSS1 facilitates the development of human cancers including breast and prostate cancers. However, in contrast to its reduced expression in many human cancers, overpression of MTSS1 was observed in hepatocellular carcinoma [11] although its physiological significance to liver cancer remains elusive.

Functionally, MTSS1 acts as a cytoskeletal scaffold protein that regulates cytoskeletal dynamics through interacting with many different proteins such as Rac, actin and actin associated proteins [12-14]. By doing so, MTSS1 increases the formation of lamellipodia, membrane ruffles, and filopodia-like structures and is also involved in promoting the disassembly of actin stress fibers. Mechanistically, MTSS1 contains a WH2 domain in its c-terminal region that preferentially interacts with ATP-bound G-actin, an active form of actin involved in polymerization. Furthermore, it has been observed that MTSS1 possesses five-folds more affinity towards ATP-bound G-actin compared to ADP-bound G-actin associated with actin monomers in the cell. Recent studies have also suggested that MTSS1 competes with the WH2 domain-containing neuronal Wiskott—Aldrich Syndrome protein (N-WASP) VCA protein for binding with G-actin [15]. Given the critical role of N-WASP in actin modeling and cytoskeleton formation [16], these findings reveal a critical role for MTSS1 in G-actin polymerization in part by inhibiting the physiological interaction between N-WASP and G-actin. In addition to interacting directly with G-actin, MTSS1 is also reported to interact with various other proteins such as the Rac GTPase, Cortactin and RPTPδ, all of which have been well-characterized as critical regulators of cell migration, invasion and cell-cell interaction [14, 16, 17]. Therefore, MTSS1 might govern various cellular processes including cellular migration or invasion in part by influencing cellular cytoskeleton. To this end, previous studies have also indicated that MTSS1 could promote the formation of dorsal ruffels in response to PDGF to result in cell shape changes. Interestingly, PDGF induces phosphorylation of MTSS1 at Tyr-397 and Tyr-398 in a Src kinase-dependent manner [18]. These findings indicated that MTSS1 is potentially involved in mediating the PDGF signaling pathway to promote actin cytoskeleton formation via the Src-related kinases [18]. However, it remains largely unclear how MTSS1 stability is physiologicaly controlled, and which upstream signaling pathway aberrantly activated in cancer cells, contributes to the reduced MTSS1 abundance frequently observed in various human cancers.

The ubiquitin proteasome system (UPS) plays an important role in the timely regulation of key cellular proteins and thereby controlling many cellular processes including cell signaling and cell cycle regulation [19]. Dysfunction of the UPS is involved in the development of many diseases including cancer [20, 21]. Three enzymes are involved in protein ubiquitination and destruction process, the ubiquitin-activating enzyme (E1), the ubiquitin-conjugating enzyme (E2) and the ubiquitin ligase (E3), respectively and the E3 ligases determine the substrate specificity of the three-step ubiquitination process [19]. The SCF^β-TRCP^ E3 ubiquitin ligase complex plays a key role in cell cycle regulation [19]. However, its exact role as a tumor suppressor or oncogene might be tissue- or cellular context-dependent as both loss of β-TRCP, and aberrant upregulation of β-TRCP, have been reported in different types of human cancers [22]. Notably, elevated levels of β-TRCP were observed in a number of cancers including pancreatic cancer [23], gastric cancer [24] and breast cancer [25]. Furthermore, consistent with a possible oncogenic role for β-TRCP in certain tissues, another study demonstrated that suppression of β-TRCP reduces prostate cancer [26]. These findings indicate that in certain tissue types, increased expression of β-TRCP may potentially lead to enhanced degradation of its substrates including possible tumor suppressors to facilitate tumorigenesis. In keeping with this notion, we report here that the tumor suppressor MTSS1 is a novel substrate of β-TRCP. In further support of our hypothesis, we have identified an evolutionally conserved phospho-degron (DSGXXS) in MTSS1 that mediates the interaction with, and subsequent ubiquitination by β-TRCP in a CKI-dependent manner. More importantly, ectopic expression of a non-degradable MTSS1 exerts stronger effects than WT-MTSS1 in suppressing tumor cell proliferation and migration. Therefore, these studies reveal the CKI/ β-TRCP signaling axis as the novel regulatory route to govern the stability of the MTSS1 tumor suppressor and that elevated CKI or β-TRCP expression might lead to accelerated destruction of MTSS1 to facilitate tumorigenesis and tumor metastasis.

## RESULTS

### MTSS1 stability is negatively regulated by the SCF^β-TRCP^ E3 ubiquitin ligase complex

**Figure 1:**
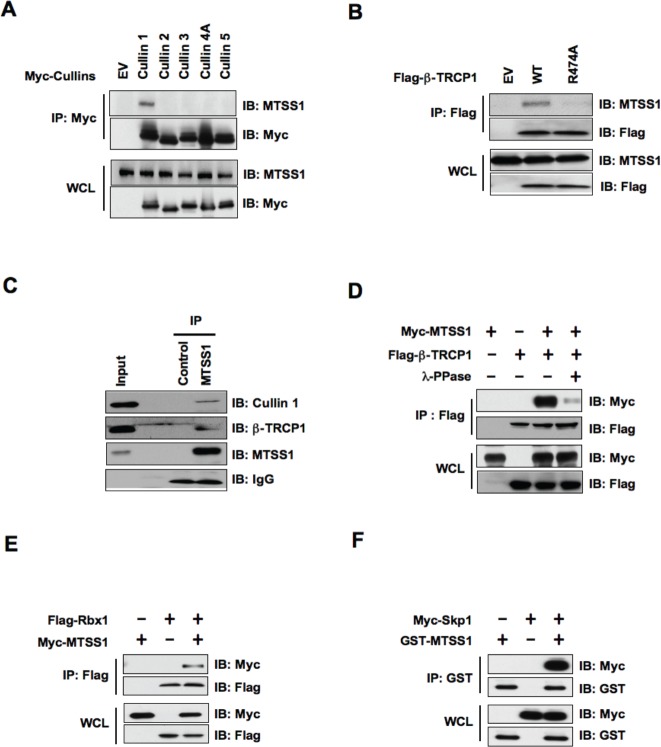
SCF complex containing β-TRCP1 and Cullin 1 interacts with MTSS1 (A) Immunoblot (IB) analysis of whole cell lysates (WCL) and immunoprecipitates (IP) derived from 293T cells transfected with Myc-tagged Cullin constructs or empty vector (EV) as a negative control. (B) IB analysis of WCL and IP derived from 293T cells transfected with Flag—tagged wild-type or R474A mutant -TRCP1 constructs, or EV as indicated. (C) 293T cell extracts were immunoprecipitated with antibody against MTSS1, or control IgG and analyzed by IB analysis. (D) IB analysis of WCL and IP derived from 293T cells transfected with Myc-MTSS1 and Flag—β-TRCP1 constructs as indicated. Where indicated, cell lysates were pre-treated with λ-phosphatase before the IP procedure. (E) IB analysis of WCL and IP derived from 293T cells transfected with Myc-MTSS1 and Flag-Rbx1 constructs, as indicated. (F) IB analysis of WCL and IP derived from 293T cells transfected with GST-MTSS1 and Myc-Skp1 constructs, as indicated.

Cullin—Ring complexes comprise the largest known class of E3 ubiquitin ligases, which play essential roles in targeting regulatory proteins for ubiquitin-mediated destruction [27]. Cullins are the critical scaffold proteins that complex with other essential components such as Skp1, F-box protein and Rbx1 to form various functional E3 ubiquitin ligases. Thus, we began our investigation by examining whether a specific Cullin—Ring complex interacts with MTSS1. Notably, we found that Cullin 1 specifically binds with endogenous as well as ectopically expressed MTSS1, but not with other members of the Cullin family (Cullin 2-5) we examined (Figure 1A and Supplementary Figure S1A). This result suggests that the SCF complex (Skp1-Cullin1-F-box protein complex), might be specifically involved in the regulation of MTSS1 protein stability. Next, we conducted studies to identify the specific F-box protein that regulate MTSS1 stability. To this end, previous studies from various groups including us showed that β-TRCP specifically binds with substrates that contain specific DSG(XX)S phosphodegron motif(s), in which the two serine residues are phosphorylated by one or more upstream kinases [28-30]. Notably, a DSG(XX)S phosphodegron motif was readily spotted within the MTSS1 protein, which was found to be conserved among various species (Figure 4A). To test the hypothesis that MTSS1 is a novel substrate of SCF^β-TRCP^, we examined whether β-TRCP directly interacts with MTSS1. We found that both exogenously expressed β-TRCP1 as well as endogenous β-TRCP1 interacts with MTSS1 (Figure 1B, 1C and Supplementary Figure S1B, S1C). Furthermore, β-TRCP1-R474A, which contains a mutation in its substrate-interacting motif [31], was deficient in associating with MTSS1, suggesting a specific interaction between β-TRCP1 and MTSS1 (Figure 1B and Supplementary Figure S1B, S1C). Importantly, we observed that phosphatase treatment significantly reduced the interaction between MTSS1 and β-TRCP1 (Figure 1D), supporting a phosphorylation-dependent interaction between MTSS1 and β-TRCP1.

**Figure 2:**
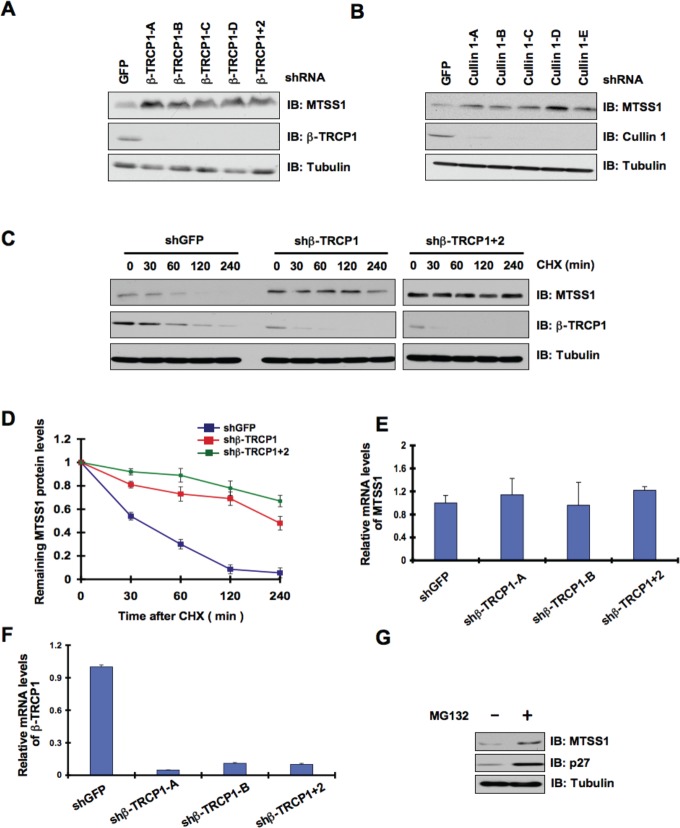
MTSS1 protein stability is controlled by the SCF^β-TRCP^E3 ubiquitin ligase (A) Immunoblot (IB) analysis of whole cell lysates (WCL) derived from 293T cells infected with shRNA constructs specific for GFP, β-TRCP1 (four independent lentiviral β-TRCP1-targeting shRNA constructs namely, -A, -B, -C, -D), or β-TRCP1+2, followed by selection with 1 μg/ml puromycin for three days to eliminate the non-infected cells. B) IB analysis of WCL from 293T cells transfected with shRNA specific for GFP, or several shRNA constructs against Cullin 1 (five independent lentiviral Cullin 1-targeting shRNA constructs namely, -A, -B, -C, -D, -E) followed by selection with 1 μg/ml puromycin for three days to eliminate the non-infected cells. (C) 293T cells were infected with the indicated shRNA constructs followed by selection with 1 μg/ml puromycin for three days to eliminate the non-infected cells. The generated stable cell lines were then split into 60-mm dishes. 20 hours later, cells were treated with 20 μg/ml CHX. At the indicated time points, WCL were prepared, and immunoblots were probed with the indicated antibodies. (D) Quantification of the band intensities in C. MTSS1 band intensity was normalized to tubulin, and then normalized to the t = 0 controls. The error bars represent mean ± SD (*n*= 3). (E-F) Relative mRNA levels of MTSS1 (E) or β-TRCP1 (F) in 293T cells infected with shRNA constructs specific for GFP, -TRCP1 (-A and -B) or β-TRCP1+2 followed by selection with 1 μg/ml puromycin for three days to eliminate the non-infected cells. MTSS1 and β-TRCP1 mRNA levels were normalized to GAPDH, and then normalized to the control cells (shGFP). (G) IB analysis of WCL derived from 293T cells treated with vehicle or MG132 as indicated.

Consistent with the key role of the SCF complex in the regulation of MTSS1 stability, we also found interactions between MTSS1 and Rbx1 (Figure 1E and Supplementary Figure S1D) as well as between MTSS1 and Skp1 (Figure 1F). These findings together suggest that the SCF complex comprising of Cullin 1, Rbx1, Skp1, and β-TRCP is involved in the regulation of MTSS1 stability. In further support of the physiological roles of β-TRCP and Cullin 1 in the regulation of MTSS1, we found that depletion of endogenous β-TRCP or Cullin 1 significantly upregulated MTSS1 (Figure 2A, 2B and Supplementary Figure S2A, S2B). Importantly, depletion of β-TRCP caused a marked increase in MTSS1 half-life (Figure 2C and 2D), but not in MTSS1 mRNA levels (Figure 2E and 2F). Moreover, in support of the notion that SCF^β-TRCP^ might regulate MTSS1 abundance in a post-translational mechanism, treatment with the proteasome inhibitor, MG132, significantly upregulated MTSS1 protein levels, indicating the potential involvement of 26S proteasome in MTSS1 degradation (Figure 2G). These findings together suggest that a post-transcriptional regulatory mechanism such as the ubiquitin proteasome system may be involved in the regulation of MTSS1 stability.

### Casein Kinase Iδ (CKIδ) is involved in the regulation of MTSS1 protein stability

**Figure 3:**
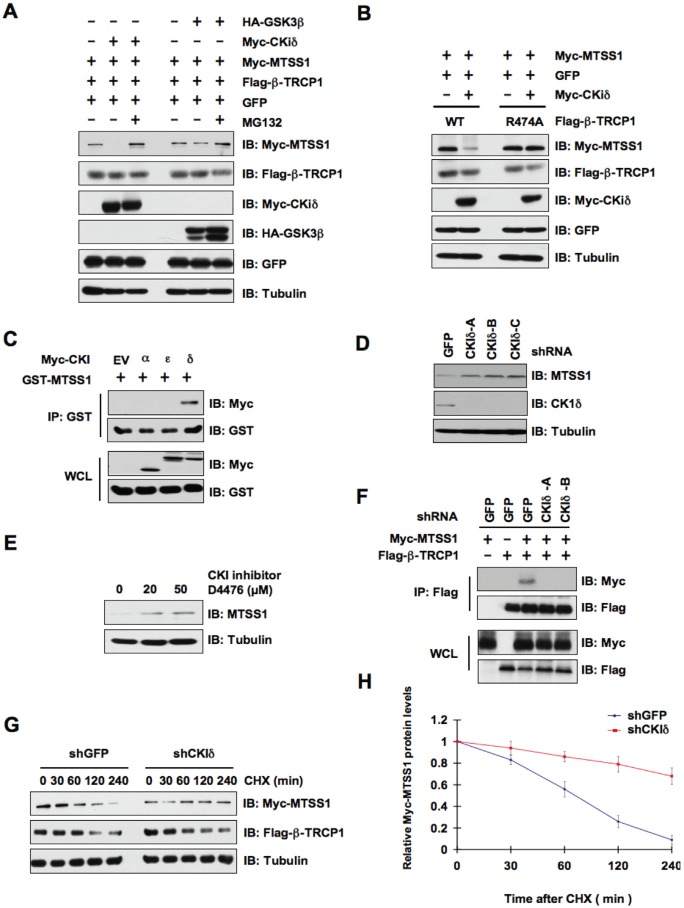
CKIδ is involved in the regulation of MTSS1 protein stability mediated by SCF^β-TRCP^ (A) Immunoblot (IB) analysis of whole cell lysates (WCL) derived from HeLa cells transfected with Myc-MTSS1, Flag-β-TRCP1, and indicated kinases. Where indicated, cells were treated with the proteasome inhibitor MG132. (B) IB analysis of WCL derived from 293T cells transfected with Myc-MTSS1 and/or Myc-CKIδ together with Flag-WT—β-TRCP1 or Flag-R474A—β-TRCP1. (C) IB analysis of WCL and immunoprecipitates (IP) derived from 293T cells transfected with GST-MTSS1 and Myc-tagged versions of the indicated CKI isoforms. (D) IB analysis of HeLa cells that were infected with shRNA specific for GFP or the indicated CKI isoforms, followed by selection with 1 μg/ml puromycin for three days to eliminate the non-infected cells. (E) IB analysis of HeLa cells treated with the CKI inhibitor D4476 at the indicated concentrations for 12 hours. (F) IB analysis of WCL and IP derived from HeLa cells that were infected with shGFP, shCKIδ-A or shCKIδ-B, followed by selection with 1 μg/ml puromycin for three days to eliminate the non-infected cells. The various generated HeLa cell lines were then transfected with Flag—β-TRCP1 and/or Myc-MTSS1 as indicated. (G) HeLa cells were infected with the indicated shRNA constructs followed by selection with 1 μg/ml puromycin for three days to eliminate the non-infected cells. The various generated HeLa cell lines were then transfected with Myc-MTSS1, Flag—β-TRCP1. 20 hours post-transfection, the cells were split into 60-mm dishes before being treated with 20 μg/ml CHX. At the indicated time points, WCL were prepared, and immunoblots were probed with the indicated antibodies. (H) Quantification of the band intensities in G. Myc-MTSS1 band intensity was normalized to tubulin, and then normalized to the t = 0 controls. The error bars represent mean ± SD (*n*= 3).

As β-TRCP only recognizes its substrates when they are properly phosphorylated by one or a combination of kinase(s) [32, 33], we sought to identify the upstream kinase that phosphorylates MTSS1 to trigger its destruction by β-TRCP. In this regard, both CKIδ and GSK3β have been previously identified as critical players in phosphorylating the targeted proteins of β-TRCP for ubiquitin-mediated protein degradation [34, 35]. Hence, to determine the specific kinase involved in the degradation of MTSS1, we transfected HeLa and 293T cells with HA-MTSS1 and Flag-β-TRCP1 along with CKIδ or GSK3β and further analyzed MTSS1 levels by western blot analysis. Interestingly, we found that CKIδ, but not GSK3β, efficiently promoted the degradation of MTSS1 (Figure 3A and Supplementary Figure S3A). Furthermore, MG132, a 26S proteasome inhibitor, completely prevented MTSS1 degradation mediated by CKIδ and β-TRCP1, suggesting the involvement of the 26S proteasome in this process (Figure 3A and Supplementary Figure S3A). Importantly, the mutant β-TRCP1 (R474A), which is unable to interact with MTSS1 (Figure 1B), failed to promote MTSS1 degradation in the presence of CKIδ (Figure 3B and Supplementary Figure S3B), indicating a critical role for the C-terminal substrate-binding WD40 repeat motif in β-TRCP1-mediated destruction of MTSS1.

To further confirm the potential role of CKIδ in MTSS1 regulation, we utilized co-immunoprecipitation (Co-IP) experiments using various Myc-tagged CKI isoforms and GST-MTSS1 to determine whether CKIδ directly interacts with MTSS1 ***in vivo***. Notably, we found that CKIδ, but not other CKI isoforms such as CKIα or CKIε, specifically interacts with MTSS1 (Figure 3C). In further support a physiological role for CKIδ in governing MTSS1 stability, we demonstrated that MTSS1 abundance was significantly elevated upon inactivation of CKIδ by either depletion of endogenous CKIδ or by using a CKI inhibitor, D4476 (Figure 3D, 3E and Supplementary Figure S3C). More importantly, either depletion of CKIδ (Figure 3F) or inactivation of CKIδ by D4476 (Supplementary Figure S3D), significantly disrupted the interaction between β-TRCP and MTSS1. In keeping with the critical role of CKIδ in MTSS1 stability control, depletion of CKIδ significantly extended the MTSS1 protein half-life (Figure 3G and 3H). These findings coherently indicated a potential role of CKIδ in negative regulation of the MTSS1 stability.

### CKIδ phosphorylates Ser322 to promote MTSS1's interaction with β-TRCP1 for subsequent ubiquitination and degradation

**Figure 4:**
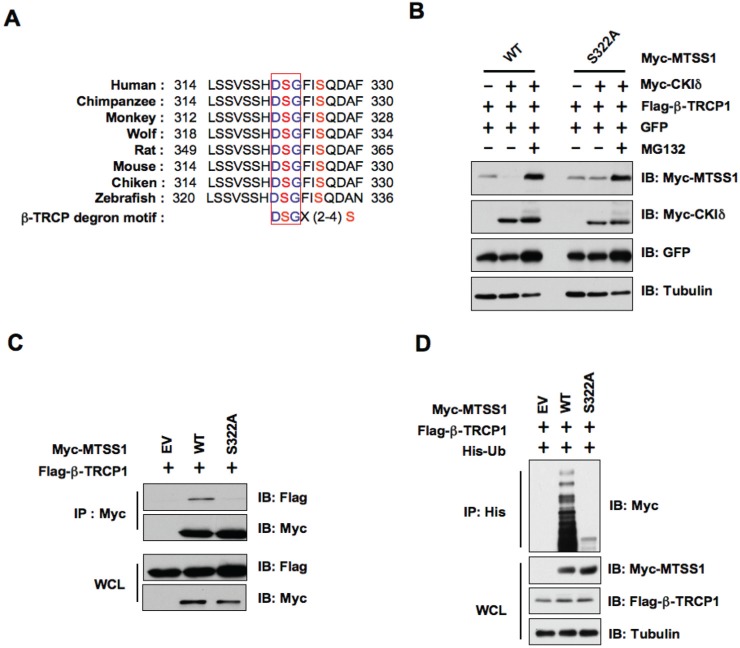
CKIδ-mediated phosphorylation of MTSS1 at Ser322 triggers its interaction with β-TRCP1 for subsequent ubiquitination and degradation (A) Alignment of the candidate phospho-degron sequence in MTSS1 from different species. (B) Immunoblot (IB) analysis of HeLa cells transfected with Flag-β-TRCP1 and Myc-tagged wild-type or S322A mutant MTSS1 constructs, as indicated. (C) IB analysis of whole cell lysates (WCL) and immunoprecipitates (IP) derived from HeLa cells transfected with Flag—β-TRCP1 together with Myc-WT—MTSS1 or Myc-S322A-MTSS1. (D) IB analysis of WCL and IP derived from 293T cells transfected with Flag-β-TRCP1, His-Ubiquitin, and Myc-tagged wild-type or S322A mutant MTSS1 constructs, or EV, as indicated.

In keeping with previously identified β-TRCP substrates, there is a canonical DSGXXS phospho-degron present in MTSS1 that could be recognized by β-TRCP upon proper phosphorylation by kinases [28]. Importantly, this degron is conserved among various species (Figure 4A). To test the significance of this putative DSGXXS phosphodegron in MTSS1 protein stability, we created a point mutation in the DSG motif by replacing the Ser322 residue with alanine (S322A). In support of the critical role for Ser322 in β-TRCP1-mediated destruction of MTSS1, we found that wild-type, but not the S322A mutant form of MTSS1, could be efficiently degraded in the presence of β-TRCP1 and CKIδ (Figure 4B). Moreover, the proteasome inhibitor MG132 completely prevented the degradation of MTSS1 suggesting the involvement of a proteasome-mediated degradation mechanism in this process (Figure 4B). In keeping with this finding, unlike WT-MTSS1, S322A-MTSS1 was deficient in interacting with β-TRCP1 (Figure 4C), providing a possible explanation for its resistance to β-TRCP1/CKIδ-mediated destruction. Consistently, ***in vivo*** ubiquitination assays revealed that wild-type, but not the S322A mutant form of MTSS1, could be ubiquitinated ***in vivo*** (Figure 4D). These findings indicated that phosphorylation of Ser322 within the canonical phospho-DSG degron motif in MTSS1 is potentially involved in governing MTSS1 destruction mediated by β-TRCP and CKIδ.

### β-TRCP-mediated destruction of MTSS1 affects cancer cell proliferation and migration

**Figure 5:**
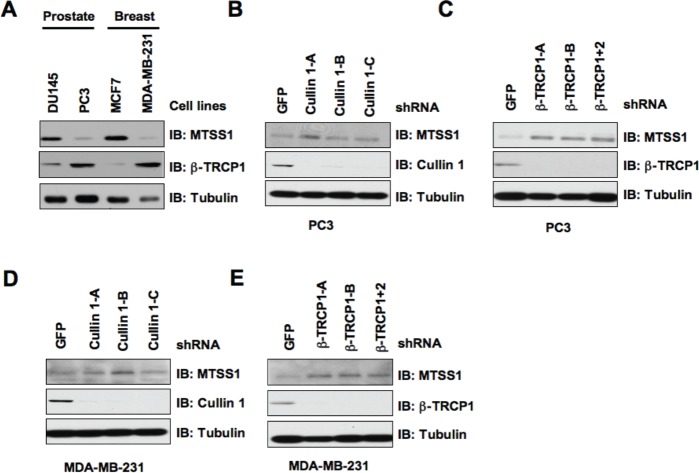
β-TRCP levels inversely correlate with MTSS1 abundance in several cancer cell lines (A) Whole cell lysates (WCL) prepared from the indicated cancer cell lines were analyzed by immunoblot (IB) analysis. (B) IB analysis of WCL prepared from PC3 cells that were infected with shRNA constructs specific for GFP or Cullin 1, followed by selection with 1 μg/ml puromycin for three days to eliminate the non-infected cells. (C) IB analysis of WCL prepared from PC3 cells that were infected with shRNA constructs specific for GFP, β-TRCP1 (A, B), or β-TRCP1+2, followed by selection with 1 μg/ml puromycin for three days to eliminate the non-infected cells. (D) IB analysis of WCL prepared from MDA-MB-231 cells that were infected with shRNA constructs specific for GFP or Cullin 1, followed by selection with 1 μg/ml puromycin for three days to eliminate the non-infected cells. (E) IB analysis of WCL prepared from MDA-MB-231 cells that were infected with shRNA constructs specific for GFP, β-TRCP1 (A, B), or β-TRCP1+2, followed by selection with 1 μg/ml puromycin for three days to eliminate the non-infected cells.

Given that a significant decrease in MTSS1 abundance is frequently observed in both prostate and breast cancers [7, 10], we sought to investigate whether MTSS1 expression in these cancer cells inversely correlates with cellular proliferation and migration. To begin this investigation, we first analyzed MTSS1 protein levels in various prostate and breast cancer cell lines. Notably, we found that the PC3 prostate cancer cells and the MDA-MB-231 breast cancer cells displayed a significantly reduced expression of MTSS1, whereas DU145 and MCF-7 cells expressed relatively high MTSS1 levels (Figure 5A). Furthermore, we noticed that the MTSS1 levels inversely correlate with the endogenous β-TRCP1 levels, arguing that β-TRCP1 expression levels might dictate the abundance of MTSS1 in this experimental setting. To further examine this hypothesis, we depleted endogenous Cullin 1 or β-TRCP via lentiviral shRNA infection to examine its effects on MTSS1 abundance. In keeping with a critical role for SCF^β-TRCP^ in governing MTSS1 stability, we found that depletion of either Cullin 1 or both β-TRCP isoforms led to a signficant upregulation of MTSS1 in both PC3 and MDA-MB-231 cells (Figure 5 B-E).

**Figure 6:**
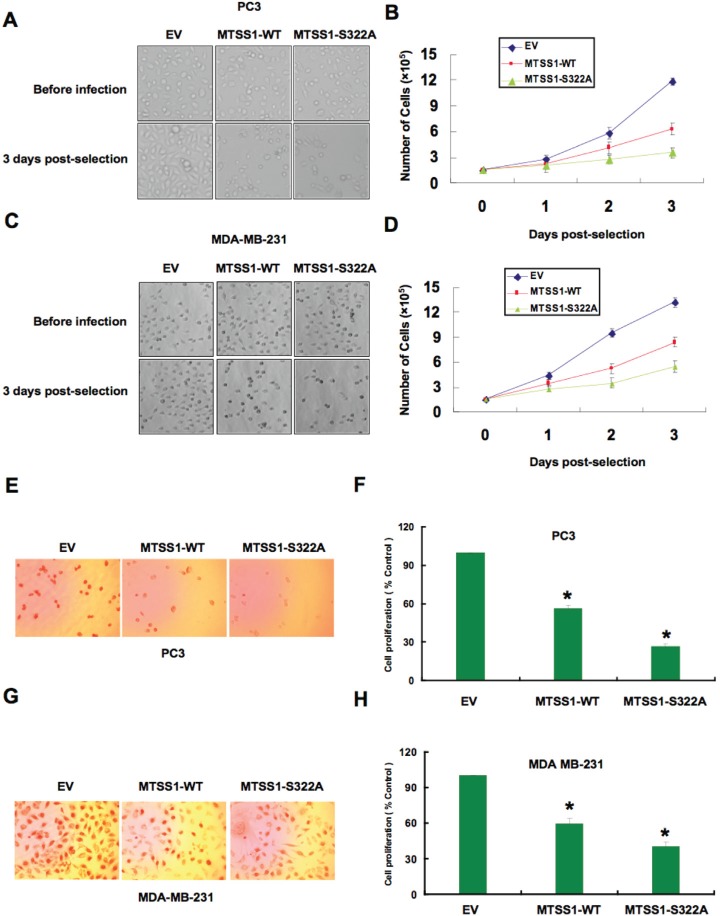
Mutant MTSS1 inhibits PC3 and MDA-MB-231 cancer cell proliferation (A-D) PC3 (A, B) or MDA-MB-231 (C, D) cells were infected with pBabe-EV, pBabe-HA-wild-type-MTSS1 or pBabe-HA-S322A-MTSS1 retroviral vectors and photographs were taken after growing the generated stable cell lines for 3 days in puromycin (1 μg/ml) selection medium to eliminate the non-infected cells. The numbers of PC3 (B) or MDA-MB-231 (D) cells were quantified at the indicated time points. The number of cells was normalized against the number of cells in the corresponding pBabe-EV cells. The error bars represent mean ± SD (*n*= 3). (E-H) PC3 (E, F) or MDA-MB-231 (G, H) cells were infected with pBabe-EV, pBabe-HA-wild-type-MTSS1 or pBabe-HA-S322A-MTSS1 retroviral vectors and photographs were taken after growing the cells for 3 days in puromycin (1 μg/ml) selection medium to eliminate the non-infected cells. Furthermore, the generated cell lines were subjected to pulse of BrdU and then immunostained using anti-BrdU antibody as described in the methods section. Quantitative measurements of PC3 (F) or MDA-MB-231 (H) cells stained for BrdU were presented. The number of cells was normalized against the number of cells in the corresponding pBabe-EV cells. The error bars represent mean ± SD (*n*= 3) * p< 0.05.

These results indicated that the SCF complex consisting of Cullin 1 and β-TRCP might play a key role in the regulation of MTSS1 in both breast and prostate cancer cells. As β-TRCP is the first identified E3 ligase for MTSS1, to explore the biological significance for SCF^β-TRCP^-mediated destruction of MTSS1, next we intended to examine how ectopic expression of a non-degradable mutant form of MTSS1 (S322A-MTSS1) or wild-type MTSS1 (as a control) in both PC3 and MDA-MB-231 cancer cells could affect cellular migration or proliferation (Supplementary Figure S4A-B). Empty vector (EV) expressing cells were also used as a negative control for this experimental system. Importantly, S322A-MTSS1 expressing PC3 and MDA-MB-231 cells exhibited significantly reduced growth potential compared to wild-type MTSS1 or EV infected cells (Figure 6A-6D). Consistent with this finding, ectopoic expression of S322A-MTSS1 exerted stronger ability than WT-MTSS1 or EV controls in decreasing cell entry into the S phase, as illustrated by reduced BrdU staining in both PC3 and MDA-MB-231 cells (Figure 6E-6H). This suggests that elevated MTSS1 expression, in part due to deficient destruction by the SCF^β-TRCP^ E3 ligase, might suppress tumorigenesis by reducing S phase entry and cellular proliferation.

**Figure 7:**
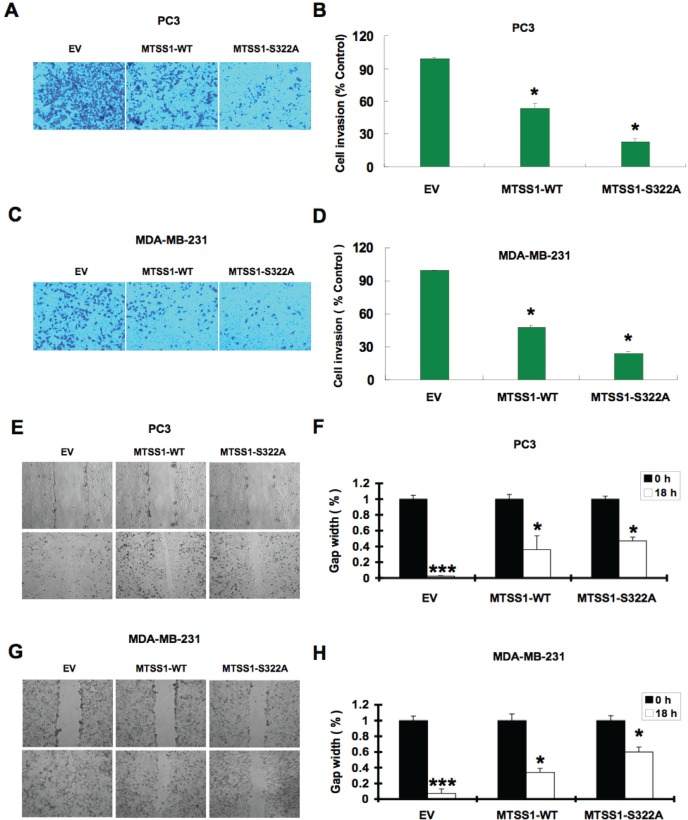
Mutant MTSS1 inhibits PC3 and MDA-MB-231 cancer cells migration (A-D) PC3 (A) or MDA-MB-231 (C) cells were infected with pBabe-EV, pBabe-HA-wild-type-MTSS1 or pBabe-HA-S322A-MTSS1 retroviral vectors and after 3 days of puromycin (1 μg/ml) selection to eliminate the non-infected cells, the generated various cell lines were subjected to trans-well cell migration assay and photographed. Quantitative measurement of migrated PC3 (B) or MDA-MB-231 (D) cells was assessed after 12 hours. The number of cells was normalized against the number of cells in the corresponding pBabe-EV cells. The error bars represent mean ± SD (*n*= 3) * p< 0.05 (n= 3). (E-F) Scratch assays were performed with PC3 cells that were infected with pBabe-EV, pBabe-HA-wild-type-MTSS1 or pBabe-HA-S322A-MTSS1 retroviral vectors followed by 3 days of puromycin (1 μg/ml) selection to eliminate the non-infected cells. The generated various PC3 cell lines were seeded on a 6 well plate and scratched on the surface with a 200 μl pipette tip. Relative values were set at 1 of the gap width at the time of the scratch. Representative photographs at time points 0 and 18 hours after the scratch (E). Measurements were done in duplicate in 3 separate experiments, and data were depicted as average gap width (F). The error bars represent mean ± SD (*n*= 3). ***p<0.001; *p<0.05. (G-H) Scratch assays were performed with MDA-MB-231 cells that were infected with pBabe-EV, pBabe-HA-wild-type-MTSS1 or pBabe-HA-S322A-MTSS1 retroviral vectors followed by 3 days of puromycin (1 μg/ml) selection to eliminate the non-infected cells. The generated various MDA-MB-231 cell lines were seeded on a 6 well plate and scratched on the surface with a 200 μl pipette tip. Relative values were set at 1 of the gap width at the time of the scratch. Representative photographs at time points 0 and 18 hours after the scratch (G). Measurements were done in duplicate in 3 separate experiments, and data were depicted as average gap width (H). The error bars represent mean ± SD (*n*= 3). ***p<0.001; *p<0.05.

**Figure 8:**
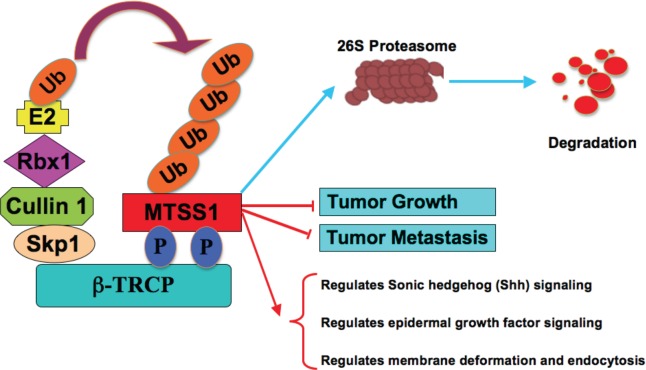
Proposed model for how aberrant elevation of SCF^β-TRCP^-mediated ubiquitination and degradation of the MTSS1 tumor suppressor may contribute to tumorigenesis and metastasis in part by promoting tumor cell growth and migration.

Furthermore, given the well-characterized role of MTSS1 in both cell cytoskeleton remodeling and cellular migration, we conducted cell migration assays to investigate how SCF^β-TRCP^—mediated destruction of MTSS1 might affect cellular migration, an important feature of human cancer invasion and metastasis. Notably, compared to wild-type MTSS1 and empty vector expressing cells, S322A-MTSS1 expressing prostate (PC3) and breast (MDA-MB-231) cancer cells exhibited a significant reduction in cell migration (Figure 7A-7D) and subsequently, reduced ability in recovering from scarring (Figure 7E-7H). Importantly, these results coherently suggest that non-degradable MTSS1 expressing cancer cells exhibit more dramatic effects in inhibiting growth and migration compared to wild-type MTSS1 or EV-expressing cells, advocating a critical role of SCF^β-TRCP^—mediated destruction of MTSS1 in suppressing tumor growth and migration (Figure 8).

## DISCUSSION

Downregulation of MTSS1 has been observed in many types of human cancers, and complete loss of MTSS1 is associated with poorly differentiated metastatic tumors, and with poor survival rate [6-9]. However, the underlying molecular mechanisms responsible for reduced expression of MTSS1 in human cancers remain largely unknown. To this end, our study, for the first time, identified the β-TRCP/CKIδ signaling as a major regulatory mechanism that governs MTSS1 degradation. β-TRCP is an F-box protein, which regulates many cellular processes by the timely targeting of various substrates for proteasome-mediated degradation [36]. However, the exact role of β-TRCP in tumorigenesis could be tissue specific or cellular context-dependent. β-TRCP dysfunction, including both loss of β-TRCP and elevated expression of β-TRCP, have been reported in distinct types of human cancers [22]. Notably, β-TRCP overexpression has been reported in certain tumor types including human breast or prostate cancers, which could lead to increased degradation of its substrates [24, 37]. Our study suggests that at least in the breast or prostate cancer settings, β-TRCP overexpression could possibly lead to increased degradation of the tumor suppressor MTSS1, thereby contributing to tumorigenesis. In this direction, we found a possible inverse relationship between MTSS1 and β-TRCP expression in both prostate and breast cancer cell lines (Figure 5A). Our study also demonstrated that β-TRCP specifically interacts with MTSS1 in a CKIδ-dependent manner, and further promotes the ubiquitination and subsequently proteasome-mediated degradation of MTSS1 (Figure 4B). Although previous reports indicate that dysregulation in MTSS1 gene expression in part contributes to loss of MTSS1 [8], our study indicates the accelerated MTSS1 degradation, possibly due to elevated expression of β-TRCP, could be an alternative mechanism accounting for reduced abundance of the MTSS1 tumor suppressor in various human cancers.

In this study, we also identified CKIδ as the upstream kinase that is potentially involved in β-TRCP-mediated degradation of MTSS1. We found that CKIδ, but not other CKI isoforms, phosphorylates MTSS1 at Ser322 within the DSGXXS phosphodegron to trigger its interaction with β-TRCP for subsequent ubiquitination and degradation of MTSS1. Consistently, wild-type, but not a S322A mutant form of MTSS1 interacted with β-TRCP. As a result, S322A-MTSS1 was resistant to β-TRCP/CKIδ-mediated degradation, thereby displaying extended half-life. These findings support a critical role for phosphorylation of Ser322 in regulating MTSS1 stability. However, it remains to be determined whether in addition to elevated β-TRCP expression [24, 37, 38], cancer cells also exhibit increased CKI activity, resulting in increased degradation of MTSS1. Notably, it has been demonstrated that aberrant CKI activity is linked to carcinogenesis [39]. More specifically, the CKIδ and CKIε isoforms have been known to possess growth promoting and anti-apoptotic characteristics, and elevated CKIδ and CKIε activities are associated with the development of ductal carcinoma of the pancreas [40]. Moreover, increased CKI activity promotes SV40-induced cellular transformation both ***in vitro*** and ***in vivo***, suggesting a potential oncogenic role of CKI in tumorigenesis [41]. Taken together, these observations indicate that aberrancies in the MTSS1 degradation pathway, either by elevated expression of β-TRCP, or hyper-activation of CKIδ, may possibly downregulate the MTSS1 tumor suppressor to facilitate tumor cell growth and cancer progression (Figure 8).

Consistent with this notion, we found that compared to cells expressing either WT-MTSS1 or EV control, ectopic expression of non-degradable MTSS1 (S322A) significantly reduced cell growth and S phase entry as evidenced by decreased BrdU staining, as well as markedly halted cellular migration ability. These findings indicate that MTSS1 expression could prevent cancer cell growth, migration and possibly invasion. Although the precise molecular mechanisms by which MTSS1 inhibits tumor growth and metastasis remain largely unknown, it was reported that MTSS1 levels inversely correlate with the growth, invasion, adhesion and migration of kidney cancer cells, and MTSS1 suppresses kidney cancer cell migration via the Sonic hedgehog (SHH) pathway [9]. Other studies have indicated that MTSS1 promotes cell-cell junction assembly through recruiting the small GTPase and actin, which drives junction maintenance. Therefore, loss of MTSS1 in cancers may lead to the loss of junction stability, which ultimately promotes EMT and metastasis [42]. Furthermore, MTSS1 is known to negatively regulate the epidermal growth factor signaling to suppress metastasis [43]. Further studies are required to reveal the exact molecular mechanisms and signaling pathways through which MTSS1 modulates cancer cell migration and invasion.

In summary, our study provides a possible novel molecular mechanism for the frequent reduction in expression of the MTSS1 tumor suppressor in various types of human cancers. Our work further suggest that in part by restoring MTSS1 expression to suppress cancer cell growth, proliferation and metastasis, β-TRCP inhibitors or CKI inhibitors may be beneficial in treating various types of human cancers, particularly the metastatic cancers that are associated with poor survival rates.

## MATERIAL METHODS

### Cell Culture

Hela, 293T, 293FT cells and the breast cancer cell lines MCF-7 and MDA-MB-231 were cultured in DMEM medium (Life Technologies, CA) supplemented with 10% FBS, penicillin and streptomycin. The prostate cancer cell lines DU145 and PC3 were cultured in RPMI 1640 medium with 10% FBS and antibiotics.

### Plasmids

MTSS1 cDNAs were subcloned using the Pfu polymerase (Agilent Technologies) into the pCMV-GST [34] or the pCDNA3-Myc vector to create GST-MTSS1 or Myc-MTSS1 in frame fusion protein, respectively. Short hairpin RNAs (shRNA lentivirus vectors), i.e., shRNA—β-TRCP1, shRNA—β-TRCP1+2, shRNA-GFP, and CKI constructs were described previously [32, 44]. Flag—β-TRCP1 and Flag—β-TRCP1-R474A constructs were described previously [30, 34]. Myc-Cullin 1, Myc-Cullin 2, Myc-Cullin 3, Myc-Cullin 4A, and Myc-Cullin 5 constructs were gifts from J. DeCaprio (Dana-Farber Cancer Institute, Boston, MA). Lentiviral shRNA constructs against GFP and CKIδ were obtained from W. Hahn (Dana-Farber Cancer Institute, Boston, MA). shRNA lentiviral vectors against Cullin 1 and Cullin 4A were gifts from J. Wade Harper (Harvard Medical School, Boston, MA).

### Cell transfection and viral transduction procedures

For cell transfection, 5 × 10^5^ HeLa or 293T cells were seeded in 60-mm plates and transfected using Lipofectamine (Invitrogen) in OptiMEM medium (Invitrogen) for 48 hours according to the manufacturer's instructions. For viral transduction experiments, 6 × 10^5^ HEK 293T cells were seeded in 60-mm dishes and cotransfected the next day with each lentivirus or retrovirus vector, along with helper plasmids (i.e., gag-pol and VSV-G were used for lentiviral infections). Media with progeny virus from transfected cells was collected every 24 h for 2 d, and then filtered with 0.45-μm filters (Millipore) and freshly used to infect 293T, HeLa, prostate or breast cancer cells overnight in the presence of 8 μg/ml Polybrene (Sigma-Aldrich). After infection, the cells were selected with 1 μg/ml puromycin (Sigma-Aldrich) for 72 hours to eliminate the uninfected cells before collecting the whole cell lysates (WCLs) for the subsequent biochemical assays. Knockdown or overexpression in the transduced cells was confirmed by real-time RT-PCR or western blot analysis.

### Antibodies and reagents

Anti-MTSS1 antibody (4386) was purchased from Cell Signaling Technology. α-c-Myc (9E10) and polyclonal α-HA antibodies (Y-11) were purchased from Santa Cruz Biotechnology. α-Tubulin antibody (T-5168), α-Vinculin antibody (V-4505), polyclonal α-Flag antibody (F-2425), monoclonal α-Flag antibody (F-3165), α-HA agarose beads (A-2095), peroxidase-conjugated α-mouse secondary antibody (A-4416) and peroxidase-conjugated α-rabbit secondary antibody (A-4914) were purchased from Sigma. Monoclonal α-HA antibody (MMS-101P) was purchased from Covance and α-GFP antibody (632380) was from Invitrogen. Anti-Cullin 1 (4995) and anti—β-TRCP1 (4394) antibodies were purchased from Cell Signaling Technology.

### Immunoblots and immunoprecipitation

Cells were lysed in EBC-lysis buffer (50 mM Tris, pH 8.0, 120 mM NaCl, and 0.5% NP-40) supplemented with protease inhibitors (Complete Mini; Roche) and phosphatase inhibitors (phosphatase inhibitor cocktail set I and II; EMD Millipore). The protein concentrations of the lysates were measured using a protein assay reagent (Bio-Rad Laboratories, CA) on a DU-800 spectrophotometer (Beckman Coulter). The lysate samples were then resolved by SDS-PAGE and immunoblotted with the indicated antibodies. For immunoprecipitation assays, 20 hrs of post transfection, cells were treated with 10 μM MG132 overnight before harvesting for immunoprecipitation. 800 μg of protein lysates were incubated with the appropriate antibodies (1—2 μg) overnight at 4˚C, followed by addition of carrier beads. Immunocomplexes were washed five times with NETN buffer (20 mM Tris, pH 8.0, 100 mM NaCl, 1 mM EDTA, and 0.5% NP-40) before being resolved by SDS-PAGE and immunoblotted with indicated antibodies.

### Protein degradation analysis and protein half-life studies

Cells were seeded in 6-cm culture dishes 20 hrs before transfection. Cells were transfected with 2.0 μg Myc-MTSS1, along with 1.0 μg Flag-β-TRCP1 and 0.1 μg of a plasmid encoding GFP as an internal control, in the presence or absence of 0.4 μg Myc-CKIδ. For half-life studies, 20 μg/ml cycloheximide (CHX; Sigma-Aldrich) was added to the medium 40 hrs of post transfection. At various time points thereafter, cells were lysed and protein concentrations were measured. 30 μg of the indicated whole cell lysates (WCL) were separated by SDS-PAGE and protein levels were measured by immunoblot analysis.

### *In vivo* ubiquitination assay

Cells were transfected with His-Ubiquitin along with Myc-MTSS1 (wild-type or S322A) and Flag-β-TRCP1. Thirty-six hours after transfection, cells were harvested, and the lysates were incubated with Ni-NTA matrices (Qiagen) at 4 ˚C for 12 h in the presence of 8 M Urea pH 7.5. Immobilized proteins were washed five times with 8 M Urea pH 6.3 before being resolved by SDS-PAGE and immunoblotted with the anti-Myc antibody [45].

### Scratch assay

Cancer cells were grown to confluency in a 6-wells plate. The cell monolayer was scraped in a straight line with a tip. Photographs of the scratch were taken at 0 h and 18 h. Gap width at 0 h was set to 1. Gap width analysis was performed with PhotoshopCS4 using the analytical ruler tool. Measurements were taken at multiple defined sites (>5) along the scratch. Each scratch was given an average of all measurements. Data are expressed as the average of three independent experiments.

### Cell migration assay

For cell migration assay, 1 × 10^5^ cells in serum-free media containing 0.1% BSA were added to the upper chamber of a Transwell Filter (8 μm pore size; Corning) in triplicates. Cell-conditioned media was added to the lower chamber. After a 16-h incubation at 37 ˚C, non-migrated cells at the top of the filter were removed using cotton swabs. Cells that had migrated to the bottom of the filter were fixed and stained using the Hema-3 stain set. Cells were then counted using a 20× objective, and four fields were chosen per well with three wells per each condition [29].

### Bromodeoxyuridine (BrdU) labeling

BrdU labeling was performed as described previously [46]. Briefly, cultures were incubated with 1 μg/ml (3 μM) BrdU and 1 mg/ml uridine for 48 h. Cells were washed with PBS, fixed with ice-cold absolute methanol for 10 min, treated with 1.5 M HCl for 1 h at room temperature, and neutralized with 0.1 M borate buffer (pH 8.5) for 30 min. After blocking with 0.1% bovine serum albumin (BSA) in PBS for 30 min at 37˚C, cells were incubated with 5 μg/ml anti-BrdU monoclonal antibody (PharMingen) in 0.1% BSA/PBS for 1 h, washed with 0.1% BSA/PBS, and incubated with 1 μg/ml HRP-conjugated rabbit anti-mouse secondary antibody (Jackson Immunoresearch) for 1 h. Cells were then washed extensively with ammonia-buffered phosphate (ABP; 0.1 M NaH_2_PO4 brought to pH 7.0 with ammonium hydroxide) and stained for 12—16 h at room temperature with 1.3 mM 3,3-diaminobenzidine in ABP containing 0.004% H_2_O_2_. The experiments were performed 3 times to generate error bars.

## Supplementary Figures


