# Supplementary material for: SCFβ-TRCP targets MTSS1 for ubiquitination-mediated destruction to regulate cancer cell proliferation and migration
Source: Oncotarget. 2013 Nov 6;4(12):2339–53. doi: 10.18632/oncotarget.1446 (PMC3926831; doi:10.18632/oncotarget.1446)
Supplement: Supplementary file 1 [file oncotarget-04-2339-s001.pdf]

SCFβ-TRCP targets MTSS1 for ubiquitination-mediated destruction to regulate cancer cell proliferation and migration - Zhong et al

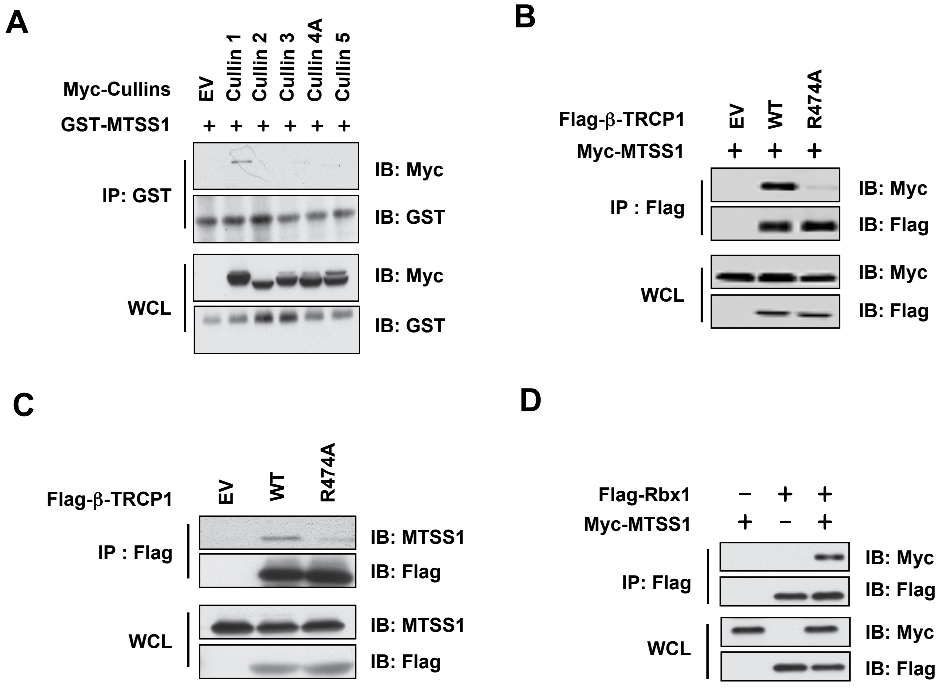

Figure S1: SCF$^{β\text{-TRCP}}$ E3 ligase complex interacts with MTSS1. (A) Immunoblot (IB) analysis of whole cell lysates (WCL) and immunoprecipitates (IP) derived from 293T cells transfected with GST-MTSS1 and Myc-tagged Cullin constructs or EV, as indicated. (B) IB analysis of WCL and IP derived from HeLa cells transfected with Myc–MTSS1 and Flag-tagged wild-type or R474A mutant β-TRCP1 constructs, or EV, as indicated. (C) IB analysis of WCL and IP derived from HeLa cells transfected with Flag–β-TRCP1 wild-type or R474A mutant constructs, or EV. (D) IB analysis of WCL and IP derived from HeLa cells transfected with Myc-MTSS1 and Flag-Rbx1 constructs.

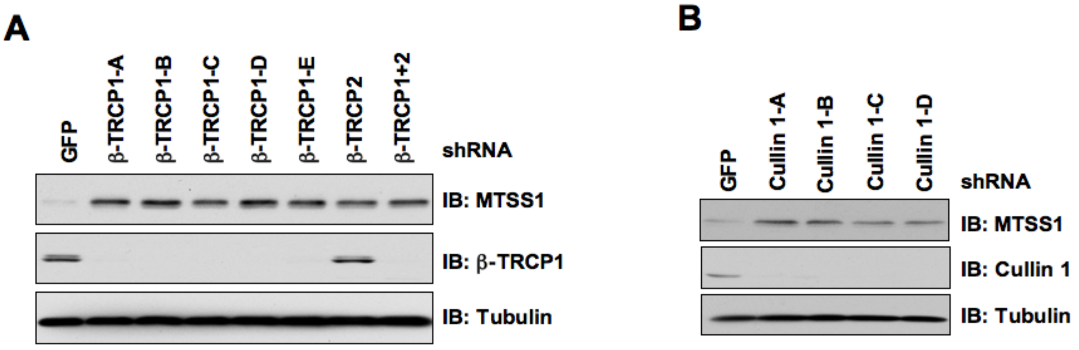

Figure S2: Depletion of endogenous β-TRCP or endogenous Cullin 1 led to a significant upregulation of MTSS1. (A) Immunoblot (IB) analysis of whole cell lysates (WCL) derived from HeLa cells that were infected with shRNA constructs specific for GFP, β-TRCP1 (five independent lentiviral β-TRCP1-targeting shRNA constructs namely, -A, -B, -C, -D, -E), -TRCP2 or β-TRCP1+2, followed by selection with 1 μg/ml puromycin for three days to eliminate the non-infected cells. (B) IB analysis of WCL derived from HeLa cells that were infected with shRNA specific for GFP, or several shRNA constructs against Cullin 1 (four independent lentiviral Cullin 1-targeting shRNA constructs namely, -A, -B, -C, -D), followed by selection with 1 μg/ml puromycin for three days to eliminate the non-infected cells.

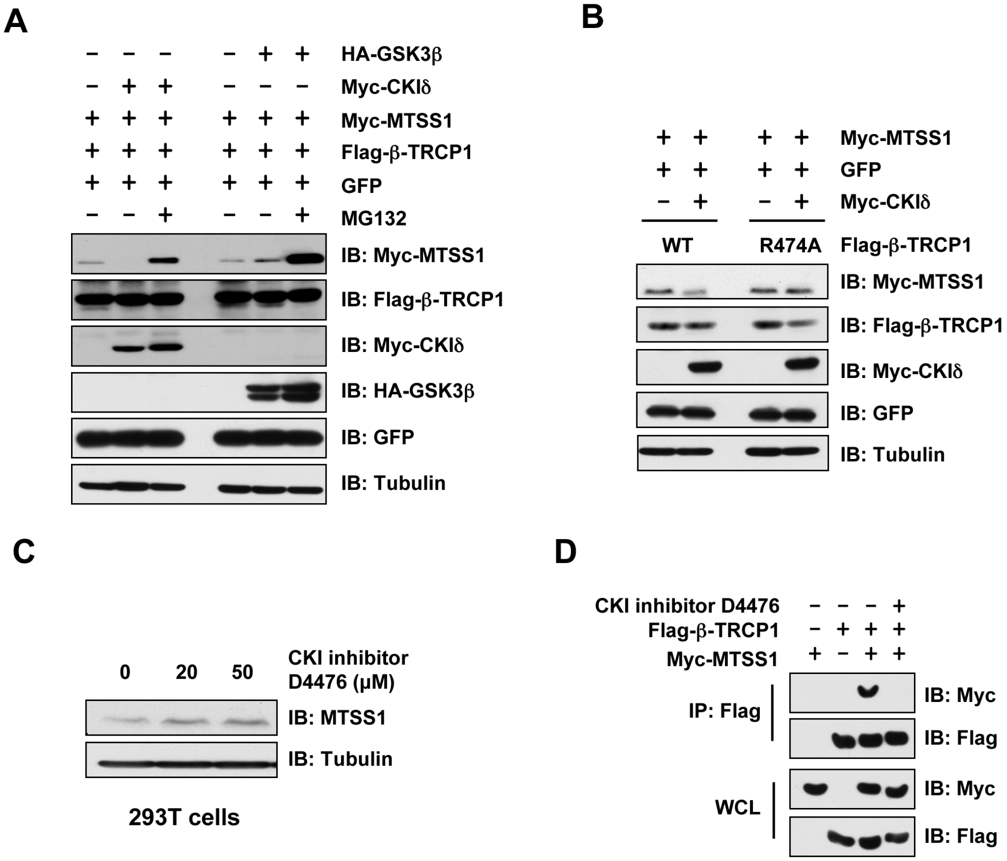

Figure S3: Depletion of endogenous β-TRCP or endogenous Cullin 1 led to a significant elevation of MTSS1 protein abundance. (A) Immunoblot (IB) analysis of whole cell lysates (WCL) derived from 293T cells transfected with Myc-MTSS1, Flag-β-TRCP1, and indicated kinases. Where indicated, cells were treated with the proteasome inhibitor MG132. (B) IB analysis of WCL derived from HeLa cells transfected with Myc-MTSS1 and/or Myc-CKIδ together with Flag-WT–β-TRCP1 or Flag-R474A–β-TRCP1. (C) IB analysis of WCL derived from 293T cells treated with the CKI inhibitor D4476 at the indicated concentrations for 12 h. (D) IB analysis of WCL derived from 293T cells transfected with Myc-MTSS1 and/or Flag-β-TRCP1, as indicated. Where indicated, cells were treated with the CKI inhibitor D4476.

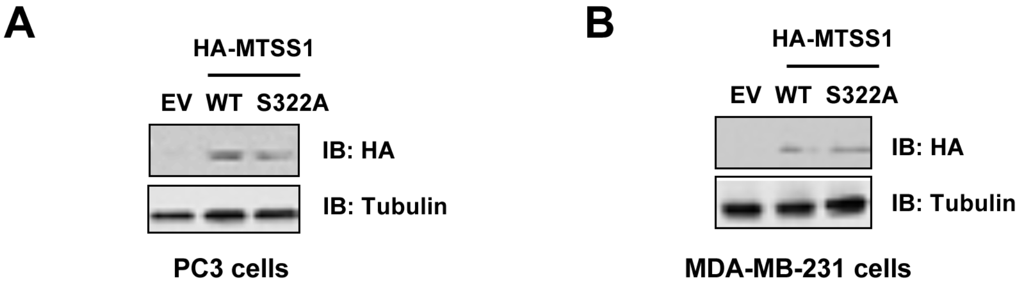

Figure S4: Generation of the PC3 and MDA-MB-231 cell lines stably expressing wild-type MTSS1 or S322A-MTSS1. (A) Immunoblot (IB) analysis of whole cell lysates (WCL) derived from PC3 cells that were infected with pBabe-EV, pBabe-HA-wild-type-MTSS1 or pBabe-HA-S322A-MTSS1 retroviral vectors, followed by 3 days of puromycin (1 μg/ml) selection to eliminate the non-infected cells. (B)Immunoblot (IB) analysis of whole cell lysates (WCL) derived from MDA-MB-231 cells that were infected with pBabe-EV, pBabe-HA-wild-type-MTSS1 or pBabe-HA-S322A-MTSS1 retroviral vectors, followed by 3 days of puromycin (1 μg/ml) selection to eliminate the non-infected cells.
